# Tuberculosis and Short Bowel: A Therapeutic Challenge

**DOI:** 10.5005/jp-journals-10071-23157

**Published:** 2019-04

**Authors:** Saurabh Mittal, Karan Madan, Nitish Aggarwal, Anita Dhar

**Affiliations:** 1,2 Department of Pulmonary, Critical Care and Sleep Medicine, All India Institute of Medical Sciences, New Delhi, India; 3,4 Department of Surgical Disciplines, All India Institute of Medical Sciences, New Delhi, India

## Abstract

**How to cite this article:** Mittal S, Madan K, *et al.* Tuberculosis and Short Bowel: A Therapeutic Challenge. Indian J Crit Care Med 2019;23(4):199.

Sir,

Tuberculosis (TB) is not an uncommon entity in the critical care setting and diagnosis requires a high index of suspicion. There is scarce data available regarding antituberculous treatment protocols in critically ill patients in intensive care unit (ICU) wherein multiple factors may contribute to drug malabsorption and issues with oral administration of these medications.^1^ Other problems associated with treatment of TB in ICU include septic shock and bowel dysfunction, which also affect drug absorption. Due to these issues, treatment of TB in critically ill patients is often challenging using the conventional treatment protocols. Among critically ill patients, only less than a third achieve therapeutic levels after administering medications through nasogastric tube.^2^ Nonavailability of intravenous formulations of conventional anti-TB drugs and lack of therapeutic drug monitoring facilities further complicates the treatment, especially in resource-limited settings. One of the difficulty to manage subgroups is patients with short bowel situation where drug absorption may be significantly affected.^3^ We herein describe our recent experience with management of one such patient.

A 19-year-old female presented to emergency department with acute abdominal pain and bleeding per rectum. She was diagnosed as acute superior mesenteric artery thrombosis with bowel necrosis. Emergency laparotomy and extensive small bowel resection were performed due to bowel necrosis. Proximal ileostomy was performed, and patient required postoperative mechanical ventilation for 3 days. Chest radiograph demonstrated bilateral alveolar infiltrates and endotracheal tube aspirate demonstrated acid-fast bacilli confirming a diagnosis of TB. Xpert MTB/RIF assay detected *Mycobacterium tuberculosis* and rifampicin resistance was not detected. Treatment with four first-line antitubercular drugs was initiated via nasogastric tube. Red colored output (likely due to rifampicin) from ileostomy loop suggested poor drug absorption. We tried feeding the dissolved tablets through distal bowel loop, but still the absorption could not be ensured as therapeutic drug level monitoring was unavailable. Patient was initiated on treatment with injectable agents linezolid, amikacin, and levofloxacin while HRZE regimen was continued. We continued same agents for 3 weeks while patient was on supplemental parenteral nutrition. She had clinical as well as radiological improvement. Afterwards, she was continued on oral drugs (HRZE with levofloxacin) for another 9 months. This regimen with more number of drugs was chosen as drug absorption was likely erratic. She had complete clinicoradiological response with therapy.

Common causes of short bowel in India include trauma, TB, Crohn's disease, vascular thrombosis, adhesive obstruction, malignancy, and multifocal bowel perforations. These patients are at high risk of malnutrition and development of TB. The intestinal absorptive function depends upon the integrity of intestinal remnant and varies whether predominantly jejunum or ileum has been resected.^4^ Jejunal resections are better tolerated as ileum can takeover absorptive function more efficiently. The drug absorption also depends upon the intactness of ileocecal junction (ICJ). In patients with intact ICJ, food stays in ileum for a longer period thus leading to better absorption. In long-term, bowel adaptation occurs leading to improved absorptive function but in acute stage drug absorption in patients with short bowel is usually poor. Another treatment option in these patients include use of higher doses of drugs, especially rifampicin and fluoroquinolones, which has been found safe.^5^ This case highlights the problem of treating TB in critically ill patients with short bowel and utility of injectable antitubercular agents in such situation.
